# ‘You say you are a TB doctor, but actually, you do not have any power’: health worker (de)motivation in the context of integrated, hospital-based tuberculosis care in eastern China

**DOI:** 10.1186/s12960-022-00745-w

**Published:** 2022-06-23

**Authors:** Guanyang Zou, Barbara McPake, Karina Kielmann

**Affiliations:** 1 School of Public Health and Management, Guangzhou University of Chinese Medicine, Guangzhou, China; 2 Nossal Institute for Global Health, University of Melbourne, Melbourne, Australia; 3 Institute for Global Health and Development, Queen Margaret University, Edinburgh, UK; 4 Department of Public Health, Institute of Tropical Medicine, Antwerp, Belgium

**Keywords:** TB health workers, Designated hospitals, Motivation, Integrated care, China

## Abstract

**Background:**

In China, tuberculosis (TB) care, traditionally provided through the Centre for Disease Control (CDC), has been integrated into ‘designated’ public hospitals at County level, with hospital staff taking on delivery of TB services supported by CDC staff. Little is known about the impact of this initiative on the hospital-based health workers who were delegated to manage TB. Drawing on a case study of two TB ‘designated’ hospitals in Zhejiang province, we explored factors influencing hospital-based health workers’ motivation in the context of integrated TB service delivery.

**Methods:**

We conducted 47 in-depth interviews with health officials, TB/hospital managers, clinicians, radiologists, laboratory staff and nurses involved in the integrated model of hospital-based TB care. Thematic analysis was used to develop and refine themes, code the data and assist in interpretation.

**Results:**

Health workers tasked with TB care in ‘designated’ hospitals perceived their professional status to be low, related to their assessment of TB treatment as lacking need for professional skills, their limited opportunities for professional development, and the social stigma surrounding TB. In both sites, the integrated TB clinics were under-staffed: health workers providing TB care reported heavy workloads, and expressed dissatisfaction with a perceived gap in their salaries compared with other clinical staff. In both sites, health workers were concerned about poor infection control and weak risk management assessment systems.

**Conclusions:**

Inadequate attention to workforce issues for TB control in China, specifically the professional status, welfare, and development as well as incentivization of infectious disease control workers has contributed to dissatisfaction and consequently poor motivation to serve TB patients within the integrated model of TB care. It is important to address the failure to motivate health workers and maximize public good-oriented TB service provision through improved government funding and attention to the professional welfare of health workers providing TB care in hospitals.

**Supplementary Information:**

The online version contains supplementary material available at 10.1186/s12960-022-00745-w.

## Background

China had the third largest tuberculosis (TB) burden in the world, with an estimated 833,000 TB patients in 2019 [1]. China’s TB control programme relies on a semi-vertical structure, operated at four levels: national, provincial, prefecture and county. TB dispensaries and the Centres for Disease Prevention and Control (CDC) at the county level have long acted as the basic TB management units providing both clinical and public health services for uncomplicated TB cases. However, it was observed that many TB patients tended to visit the general hospitals at the onset of their symptoms. Fewer cases were reported and referred to the CDC [2], which had more limited staff and clinical capacity. Since the early 2000s, an integrated service delivery model was launched by the local health bureaus, whereby selected general hospitals were designated to undertake standardized diagnosis, prescription and follow-up management of TB cases in the County catchment area. TB clinics were established within the hospital and equipped with one or two doctors and a few additional staff members, for example, nurses, pharmacists and laboratory staff. Depending on the local situation, these staff were either fixed or delegated from the infectious disease control and other departments. In this integrated model, TB care is provided by clinicians based in the designated hospital, however, public health doctors based in the CDC provide technical support and supervision of the care provided by designated hospital clinicians.

Public hospitals in China have long suffered from limited direct government funding since financial decentralization in the 1980s, with low levels of annual funding (around 12.6% of the hospitals' disposable revenue between 2002 and 2017), to cover health worker salaries and other operational costs [3]. They have instead relied heavily on user fees charged at the time of service use, but not fully, reimbursed through government subsidized health insurance. Standardized TB diagnosis and treatment is free in China. However, the government only covers outpatient-based diagnostic services and TB drugs recommended by the national guidelines, with little input to cover the operational costs of TB clinics. This has raised concerns about cost recovery for the daily operation of the TB clinics in the designated hospitals.

Drawing on qualitative interviews conducted in the context of a larger study investigating the prescribing behaviour of TB doctors in the context of integrated TB service delivery [4], this paper examines how changes in the allocation of work tasks and working relationships brought about through the integrated model of TB care affected health worker morale and motivation in these hospitals. Motivation is a key factor for health worker retention and turnover, and both a driver and a consequence of health worker performance, professional competence and quality of care outcomes [5, 6]. Motivation can be defined as ‘an individual’s degree of willingness to exert and maintain an effort towards attaining organisational goals’ [7]. It consists of a set of psychological and transactional processes resulting from the interactions between individuals and their work environment [7, 8]. Financial incentives are commonly understood to be important factors in motivating health workers [9–11], however, many studies show that financial incentives alone are insufficient: it is important to consider how enabling and conversely, disabling, working environments can affect health worker motivation. Governance, leadership and management processes are critical: studies have looked specifically at the role of supportive supervision [12, 13], performance management, evaluation and feedback [14] concern for workers’ welfare by hospital management [15], as well as resource availability [14]. While a number of studies have focused on the importance of social identity, respect, and community support for community health workers [16–18], the study of social and professional identity and respect among front-line workers in low- and middle-income hospital settings is limited [19].

Studies in China have identified a mixture of financial and non-financial incentives that affect health worker motivation and satisfaction with their working conditions. Some suggest that income, career development, and trust between fellow workers, patients and the public are the most important drivers of health worker motivation in China [10, 20]. One study described Chinese physicians as feeling ‘victimized’, ‘disenfranchised’, and ‘under-valued’ in the context of the marketization of medical care [21]. Infectious disease physicians have been specifically highlighted as facing heavy workloads, high stress levels, poor compensation and limited career prospects in China [22].

Despite these recent studies on health worker motivation in China, there is limited discussion of the specific experiences of health workers delivering care in the context of newly emerging models of integrated service delivery. This is surprising, given that ‘integration’ of what was generally perceived to be public health work into hospital-based care introduced changes in the division of labour, scope of work, and professional duties of the health workers involved.

## Methods

### Study context

The paper draws on a sub-set of qualitative data collected within the lead author’s doctoral thesis project that applied systems-based thinking to understand the prescribing behaviour of TB doctors in the context of integrated TB care in designated hospitals [4]. This was an observational and cross-sectional study conducted between 2014 and 2015 in Zhejiang Province, located in Eastern China. We selected two designated hospitals mainly based on the socio-economic and demographic features (population, per capita GDP, TB prevalence, and size of designated hospitals) of the counties where they were located, referred to hereafter as County A and County B. At the time of the study, both counties had been implementing the integrated approach to TB service delivery for a few years (Table 1, see Table 2 for a comparison of the two hospitals including the TB clinics and Box 1 for the governance and funding situation of the study hospitals). In the broader project, we used a simple systems-based framework to conceptualize the influences on doctors’ prescribing behaviour for TB as a set of dynamic relationships across health system development and financing, governance, resources, and culture [4]. In this paper, we present themes emerging in the context of this work that relate to hospital-based health workers’ experience of taking on TB care at the designated hospitals.

**Table 1:** General information of the study sites

	County A	County B
County socio-economic information		
Average GDP per capita (RMB)	34,087	31,800
Average urban resident income (RMB)	35,161	36,525
Average rural residents income (RMB)	16,938	17,023
Population	964,900	131,900
Geography	Mountain	Mountain, coastal
County health system information		
% of total government spending on health	9.9	7.3
Average government health spending per capita (RMB)	549	328
Number of health facilities	554, including 15 hospitals	695, including 15 hospitals
Number of doctors per 10,000 population	21	19.7

Estimated from the local government communiques on economic and social development in 2015 (1 USD = 6.3 RMB approximately)

**Table 2:** Basic information of study hospitals

	County A	County B
Hospital information		
Hospital staff	478	983
Hospital beds	300	600
Hospital rating^a^	2A (secondary level A)	3B (tertiary level B)
TB unit information		
TB unit setup time	2009	2011
TB unit staff	7 (2 doctors, 2 nurses, 1 pharmacist, 1 laboratory staff, 1 other)	7 (2 doctors, 2 nurses, 1 pharmacist, 1 laboratory staff, 1 other)
TB unit affiliation	Public health department	Infectious disease control department
Number of TB cases	763	700
Received GFATM support before this study	Yes	No

^a^Class III normally refers to tertiary level and Class II refers to secondary level and A and B refers to higher and lower grades, respectively

Box 1 Governance and funding situation of the study hospitals

- Clinical TB services provided within County-level designated hospitals received technical supervision and support from the CDC, both under the leadership of the health bureau.
- The government budget of the designated hospitals in County A and County B were 12% and 5% of the annual total income, respectively, in 2014.
- The designated hospital in County A received limited government funding under the budget heading of supporting the TB/public health services for the first few years of integration.
- Both hospitals received funds in line with the free treatment policy from the CDCs to cover the essential medicine and tests for standardized TB diagnosis and treatment.

### Study design, sampling, and approach

We adopted an exploratory case study approach [23] to be able to undertake an in-depth and holistic analysis drawing on the health worker interviews, observations, informal conversations, field notes and medical records of the TB patients. In this study, a ‘designated hospital’ was defined as a ‘case’.

Our study population included health workers involved in any aspect of TB service delivery in the two hospitals. Potential interviewees were identified based on their relevance to the topic of integrated service delivery and the lead author’s familiarity and previous work with the TB control programme in the respective sites. Interviews were conducted by the lead author, a Chinese male researcher working on TB control and health systems. The current paper stems from analysis of data collected during fieldwork for his Ph.D. thesis, which was supervised by the two co-authors. Through his previous work, he was able to ask CDC staff to act as gatekeepers and liaison persons, a connection that facilitated access to the hospital health workers, as the CDC is the focal point for public health management including tuberculosis, in the county.

A purposive sampling technique was used to capture a wide range of perspectives and diversity in experience relating to the integrated service delivery [24, 25]; this was complemented by a snow-balling approach to identify further interviewees, as data were being collected and new questions emerged. Recruitment of interviewees stopped at the point of data saturation [26]. All participants invited agreed to be interviewed. A total of 47 interviews were conducted, covering individuals working in the county health bureaus, CDCs and in the two counties (Table 3, Additional files 1 and 2).

**Table 3:** Categories of interviewees

Categories of interviewees	Interviewees	Number in County A	Number in County B
Health bureau staff	Vice director and disease control officer	2 (female: 0, male: 2)	2 (female: 1, male: 1)
CDC staff	Vice director and TB control officer	3 (female: 0, male: 3)	4 (female: 0, male: 4)
Designated hospital staff	Vice director, head of public health, head of medical affairs, head of accounting and financing, TB clinicians, radiologists, laboratory staff and nurses	18 (female: 7, male: 11)	18 (female: 13, male: 5)

### Data collection

Drawn from the larger project that this study is part of, the topic guide questions were focused on prescribing practice, governance, resources and culture and were adapted to the expertise of the interviewees at the time of conducting the interviews (Additional file 3). Informed oral consent was sought from the interviewees; they were informed about the purpose of the study and reassured of the confidentiality of the research and the anonymization of all aspects of their identity. The participants attended the interviews at a given time and in suitable venues, such as a vacant office, meeting room or garden; wherever possible we made sure the interviews were conducted one-on-one. Interviews were conducted face-to-face and lasted between 20 and 60 min depending on the roles of the interviewees and the depth of information that they offered. Interviews were recorded with the consent of the interviewees and transcribed verbatim. Interview notes and transcripts were anonymized following return from the field.

### Data analysis

Trustworthiness of the qualitative data was supported by following a clear study protocol, systematic data collection, and triangulation of the interview results with the informal observations. Further, extensive discussion and rigorous quality checks within the team of authors (through the doctoral supervision process) ensured critical reflection on the lead researcher’s subjectivity and position in the field (Additional file 4).

We used thematic analysis [27] to develop and refine themes, code the data and assist in interpretation, with help of Nvivo 9 [28]. The lead author read through the interview topic guides and Mandarin transcripts of the interviews and wrote up ‘thick descriptions’ of a number of interviews in each study site [29]. A thematic framework related to health worker motivation (see Table 4), mainly drawn from the resource dimension of the conceptual framework, was progressively established within the broader project framework [4]. Translation to English was only conducted after coding to maintain the original meaning and ‘flavour’ of the interviews and to ensure the accuracy of the coding. Retrieved data were further organized after constant comparison between different sub-themes. Thematic coding was conducted by the lead author and reviewed by the research team. Key findings from the overall project, including the issues raised in this paper regarding health worker motivation in the context of integrated TB services were fed back to CDC and hospital leader stakeholders during follow-up visits.

**Table 4:** Examples of themes and thick descriptions

Themes and sub-themes	Examples of thick descriptions	Examples of quotations
Professional status, discrimination and development	Perceived their professional identity as being low	‘You say you are a TB doctor, but actually, you do not have any power. For other doctors, if patients ask for treatment, doctors will have good faces. But for TB patients, most are poor, vulnerable, had little education.’ Dr. F1 (Vice Director, County A hospital)
Working conditions: dissatisfaction with payment	Unified implementation of performance-based payment and ‘economic assessment’ among all the clinical departments regarded as being unfair to the infectious disease control doctors	‘Our economic assessments system is the same as that in other clinical departments, but when there is emerging infectious disease, they say you need to do this and do that! We have become ‘superman’. There is not any preferential policy…’ Dr. Q2 (Head of Infectious Disease Control Department, County B hospital)
Working environment: concerns about infection risk and health protection	TB doctors or laboratory staff who need to have face-to-face contact with patients or sputum samples remained at most risk	‘The most important issue is the infectious risk due to the face-to-face communication between TB doctors and patients. They need to talk a lot with patients, as patients will not understand if you just explain once. It is also dangerous for the laboratory staff. So TB doctors and laboratory staff are the most dangerous.’ Dr. S2 (Head of the Central laboratory Department, County B hospital)

## Results

To provide context, we first describe informants’ perspectives on the integration of TB services in the designated hospitals before presenting emerging themes related to health worker motivation in the context of integrated TB service delivery in the designated hospitals. These are organized under three key areas: (1) professional status and identity; (2) working conditions and (3) the work environment in TB clinics of the designated hospitals.

### Integration of TB services within the designated hospitals

Informants had different perspectives on the rationale for integrating TB services within the hospital, and the associated advantages and disadvantages. In both counties, health authorities from the upper administrative levels (provincial and prefecture) had significant influence on the process. There was strong consensus between the CDC and Health Bureau that TB services should be integrated into the general hospital, given the CDC limited space and clinical capacity to manage complicated cases of TB patients.

However, views of the hospital management in both sites were somewhat more ambivalent. Hospital leaders in County A were initially hesitant about the prospect of integrating TB services due to concerns about limited resources and the impact of additional service provision on an already busy routine workload. Gradually, given the pressures towards integrating TB services at the provincial level, the hospital management became more receptive towards the idea of the integrated model which combined strengths of the hospital and CDC in TB control.

In County B, there was similar strong initial resistance from the hospital managers, due to the perceived pressures of dealing with an infectious disease and complicated public health procedures. Hospital management made the case that their infrastructure and staff were not ready to take up the new service. After considerable pressure to accept responsibility for TB treatment services and numerous communications between the health bureau, CDC and hospital, the TB clinic was set up in the infectious disease control department.

This ambivalence around integrating TB services within the hospitals in both County A and County B was reflected in the views of the health workers tasked with providing TB care under the new model.

### Professional status and identity

In County A, staff to deliver TB services were either recruited from other health facilities or shifted from other departments of the hospital and placed in a relatively independent outpatient clinic. In County B, doctors were sent by the infectious disease control department of the hospital, with regular staff rotation from various departments of the hospital.

The doctors allocated to TB care perceived their professional status as being compromised through working in the TB clinic, which was considered to be ‘second-rate’ as compared with other clinical settings. TB, a stigmatized condition associated with poverty, is associated with public health work, and therefore potentially disempowering for a clinician, as is hinted in the comments of Dr. F1 (Vice Director, County A hospital):

‘You say you are a TB doctor, but actually, you do not have any power. For other doctors, if patients ask for treatment, doctors will have good faces. But for TB patients, most are poor, vulnerable, have little education.’ [‘Face’ is a common colloquial expression in China, to indicate identity, dignity, pride].

Placing this comment in context is important: working in TB is also associated with lower income for doctors, as TB falls under the ‘free care’ directives, and infectious disease control doctors have limited capacity to generate income. Treatment of infectious diseases requires extensive use of antibiotics and antiviral medicines, however, the drug prescription control and zero-price mark-up policies that aim to counteract potential perverse incentives have reduced income generating opportunities. In contrast, in other clinical departments, income was generated from revenues gained from high-tech diagnostic tests and examinations.

Health workers working in the integrated TB clinics generally expressed a sense of professional discrimination. They felt they had been ‘misplaced’ in the TB clinic, as the hospital could not find a more ‘suitable’ post for them. Further, TB-related stigma was perceived to affect their social connections and family lives. A male TB clinic laboratory worker, in County A Hospital, commented:

‘If I say we are working in infectious disease, nobody will be happy to marry me...I feel depressed about working here.[…] it is not good for a man to keep working on TB. You just dare not tell your friends what you are doing.’

In County B, similarly, the Head of the TB Control Department of the CDC complained that: ‘Compared to doctors from other departments, TB doctors are discriminated against for treating TB. […] after all, TB is an infectious disease.’

Many felt that TB staff working in the smaller designated hospitals had limited promotion opportunities compared to those working in the higher-level hospitals. TB doctors in both hospitals could receive updated training on TB diagnosis and treatment. However, interviews with public health officials suggested that TB treatment was seen as mundane, requiring rigid adherence to simple and repetitive clinical knowledge and skills, and preventing the development of advanced medical knowledge in routine practice. Dr. A1 (health official, County A Health Bureau) shared his views:

‘Working in the TB clinic will not help to improve their medical knowledge, but will decrease their capacity as they are not exposed to the treatment of other diseases. TB is just such a small thing. If they see TB every day, it is too monotonous for them.’

### Working conditions

The perceived low status of TB health workers was underpinned by their experiences of working conditions and relationships.

#### Staff shortage and workload

The allocation of doctors to the TB clinic was dependent on the local prioritization of TB control and resources available in the designated hospitals. TB clinics were commonly only staffed with one or two doctors. In County A, two doctors shared the workload and saw approximately 40 to 50 patients per day. According to them, employing two doctors was ‘wasting resources’, in view of the current TB burden, since most of the patients just came back for routine checks and to renew their prescriptions. In County B, where the TB clinic was integrated with the infectious disease control department, the interviewees reported a serious shortage of health staff. In the infectious disease control department, there were eight doctors and eight nurses. These health workers were mainly based in the wards, but also provided consultations in a number of infectious disease clinics, including the TB clinic. There was only one doctor based in the ‘rotational’ TB clinic, Dr. M2, who complained, ‘I am exhausted, and do not have enough of a break throughout the year.’ Dr. E2, Head of TB Control Department, County B CDC agreed that ‘TB doctors are too few, and the TB clinic is very busy’.

Others confirmed that working in the TB clinic was not desirable, but that in some cases preferable in terms of the timing of work:

‘To be honest, nobody is willing to work here. I have no choice because I don’t want to take the night duties…Here in the TB clinic (generally opening from 8 a.m. to 7 p.m. during the weekdays), it is busy but not necessary to take the night duty … at least I can have a comfortable sleep at home.’ (Dr. M2, TB doctor, TB clinic, County B hospital).

#### Dissatisfaction with payment

Hospital staff are paid a basic salary based on the professional titles as specified by the government, as well as the performance-based payment, which is based on the number of patients treated and income generated from the services. Performance payment could thus vary significantly and cause a sense of inequity in staff. In County A, the TB staff were dissatisfied to receive the same bonus as the logistical staff. The bonus given to staff in other clinical departments could be several times higher than that of the TB staff since they could generate more income from treating other diseases.

In County B, similarly, staff of the infectious disease control department resented the fact that their salaries did not reflect the high workload of TB service provision, or the perceived high infectious risk. As Q2 (Director of Infectious Disease Control Department, County B hospital) noted, ‘Before TB was integrated here, we had this number of staff, but since integration, our workload has increased, but income not. Why?’.

In County B, the TB clinic, as part of the infectious disease control department, could not escape the influences and practices of ‘independent accounting’, where the department should be responsible for the income generation and general expenses of the department. Subsequently, TB doctors’ income was related to their performance with respect to income generation. In general, the unified implementation of performance-based payment and ‘economic assessment’ among all the clinical departments was regarded as putting the infectious disease control department in an unfair position, with the potential loss of income, infection risks and increasing public health tasks and emergencies. Health workers particularly noted the discrepancy between payment and compensation for working in an environment perceived to put them at high exposure to infection risks.

### Work environment

In both hospitals, the potentially high infectious disease risk and other health concerns were important factors discouraging health workers from working in the TB clinics. For example, one of the doctors in the TB clinic in County A hospital recounted how his prospective father-in-law was reluctant for him to marry his daughter due to the perceived risk of TB infection. Some health workers thought that their immune systems could be weakened if they continued to work in the TB clinic. In County B, a number of interviewees saw TB doctors or laboratory staff who had face-to-face contact with patients or sputum samples as being significantly at risk of contracting TB.

In both hospitals, however, not all health workers were concerned with the risk of infection. As a nurse in County B pointed out, working in TB clinics helped to improve their awareness of the potential risk, due to the physical existence of the TB clinic and patients wearing facemasks, as compared to working in the non-TB clinics where the infection risk was hidden. Nonetheless, the lack of risk assessment and protective measures remained a concern for TB control in both hospitals. For instance, professional facemasks like N95 were reported to have limited availability due to the high cost at the time of study.

Retaining staff to work in the integrated TB clinics was challenging, due to the perceived losses in professional identity and payment, and heightened risk of infection. As Dr. D1 (Head of TB Department of the CDC, County A) summarized: ‘It is a common problem in China. Health workers are generally unwilling to work for TB control.’

## Discussion

This study addresses the neglected topic of how the integration of infectious disease control services within a hospital context in Eastern China affected health worker conditions, relations and subsequently motivation. Our study indicates some resistance to integration of TB services in the general hospitals, which reflects the weaknesses of the financing arrangements to support integration of public health services. This research reveals how low professional self-esteem, limited opportunities for professional development and perceived stigma and risk and poor payment have resulted in poor motivation to provide TB care in the context of integrated service delivery. This is an important issue to be addressed in the health policy agenda to support the implementation of ‘The End TB Strategy’ by 2030 [30].

Our study indicates that the perceived low professional status of those working in TB may be related to weak occupational health protection and lack of financial incentives received, as described in other studies conducted in China indicating heavy workloads, high stress levels and limited career prospects among infectious disease control workers more generally [22] and expressions of feeling under-valued and vulnerable due to the marketization of medical care [21].

We also note other aspects of professional identity including the perceived stigma of working in TB firstly because of the nature of TB as an infectious disease; secondly, its association with poverty, and thirdly, because of the perception that managing TB treatment requires limited competence and skills. While TB health workers had concerns about being discriminated against because of working with infectious diseases, many other studies have suggested the social and cultural origin and influences of stigma for TB lie with TB control and treatment [31, 32]. The association of TB with poverty and consequently diminished professional power may reflect the lower status ascribed to public health work in the hospital, due to association with low pay and limited income generation ability as well as the lack of legal and political power more generally [33, 34]. However, this may also reflect the hierarchical and highly marketized medical culture [35], where wealth is a symbol of pride and identity. Hospital clinicians’ concerns with a compromised professional status can also be situated within the erosion of trust in medical professionals more generally, as reflected in widespread reports of patient-health worker disputes in China [36].

Our study indicates poor satisfaction with payment among TB health workers, resonating with other studies [11, 15, 37]. In both cases, the bonus for the TB staff still relies on the total revenue of the hospital and/or department available for distribution. The income generation-based culture of bonus allocation, combined with the independent accounting principle that disaggregates bonus calculation to the individual department creates inequities placing TB and other infectious disease control workers in a disadvantaged position. With reliance on market forces, each department of the public hospitals in China must deal with the organizational pressure to raise economic performance, causing the erosion of clinical autonomy [38]. As our earlier study suggests [39], as long as the bonus system exists, and there is insufficient government input to cover the health workers’ payment and other operational costs of the newly integrated public health services, there will continue to be pressure and distorted incentives to make profits from the patients.

We identified high levels of perceived risk of TB infection among those working in the TB clinics, which coincided with the lack of occupational risk management and protective measures, as further sources of demotivation for health workers allocated to the TB clinic. Similar barriers were identified in another study in China [40] and other LMIC settings, including the low supply of N95 particulate respirators [41].

Our study presents a classic case of market forces failing to promote public goods [42]. As TB control is a public health measure, it generates minimum income for the hospital; in hospitals where performance-based incentives prevail, the provision of TB care fails to offer incentives or benefits for health workers such as adequate payment, health and safety measures, and opportunities for professional development that might contribute to enhanced professional status. This problem undermines the Chinese health system across its many areas of function: the blind belief in localized market-based incentives means that the attempt to serve the general public good is an uphill battle that it is losing on many fronts. While the insurance system operates on the demand side to protect against the worst effects of marketization, this research underlines that protective mechanisms on the supply side have not been adequately considered.

## Limitations

We did not set out to study health worker motivation, but rather were interested to understand the rationales of prescribing practices in the context of integrating TB care within designated hospitals. As a strong emerging theme, health workers’ dissatisfaction with their new roles (and lack of associated ‘rewards’) in TB service delivery warranted more specific analysis to fill gaps in the understanding of how integrated service delivery initiatives are experienced by front-line health workers. Experiences from the two sites could have limited generalizability within China. However, using the two hospitals as examples, this study illustrates the factors influencing the job motivation of TB health workers, in the context of integrated service delivery in China. As the lead author of the paper had worked closely with the CDC in conducting operational research for TB control in China for a long time, we were aware that this might impact on his views and interpretation of the subject matter. However, we saw this connection as an advantage, as it helped us establish trust relations and gain open and frank perspectives on the status and working conditions of TB health workers in this context.

## Conclusion

In the hospitals studied, health workers’ motivation for TB care was low and stability of the workforce for TB services was consequently compromised. Inadequate attention to workforce issues for TB control in China, specifically the professional status, welfare, and development as well as incentivization of infectious disease control workers has contributed to dissatisfaction and consequently poor motivation to serve TB patients within the integrated model of TB care. It is important to recognize the conflict between the use of market forces and mechanisms to deliver public hospital services and the successful delivery of public good-oriented TB service. These tensions can probably not be reconciled without improved government funding and attention to the professional welfare of health workers providing TB care in hospitals.

## Supplementary Information

**Additional file 1.** List of interviewees in County A and County B (County Health Bureau & CDC).

**Additional file 2.** List of interviewees in County A and County B (County Designated hospital).

**Additional file 3.** Examples of interview topic guides.

**Additional file 4.** COREQ (COnsolidated criteria for REporting Qualitative research) checklist.

## Data Availability

The datasets generated and/or analysed during the current study are not publicly available due to the sensitivity of the data collected from the local health systems, but may be made available by contacting the corresponding author on reasonable request.

## Abbreviations

TB: Tuberculosis
CDC: Centres for Disease Prevention and Control
LMIC: Low- and middle-income country
GDP: Gross domestic production
