# Supplementary material for: ‘You say you are a TB doctor, but actually, you do not have any power’: health worker (de)motivation in the context of integrated, hospital-based tuberculosis care in eastern China
Source: Hum Resour Health. 2022 Jun 23;20:55. doi: 10.1186/s12960-022-00745-w (PMC9229519; doi:10.1186/s12960-022-00745-w)
Supplement: Supplementary file 1 — Additional file 1. List of interviewees in County A and County B (County Health Bureau & CDC). [file 12960_2022_745_MOESM1_ESM.docx]

Additional file 1 List of interviewees in County A and County B（County Health Bureau& CDC）

| **Organisations** | **County A** | | **County B** | |
| --- | --- | --- | --- | --- |
|  | [**Pseudonym**](http://www.baidu.com/link?url=iGB0zWxS1Oos-FNfoBOWxCiQuZRwRklCrc7VyfMl10S52ADP2lYJaUZ5KL4UhBjWVRi3dADOaEDWujNcqJIiyqkvbfDF1M8jcdMZauj3KoK&wd=&eqid=d924b88500039398000000065aa492d5) | **Basic profiles** | [**Pseudonym**](http://www.baidu.com/link?url=iGB0zWxS1Oos-FNfoBOWxCiQuZRwRklCrc7VyfMl10S52ADP2lYJaUZ5KL4UhBjWVRi3dADOaEDWujNcqJIiyqkvbfDF1M8jcdMZauj3KoK&wd=&eqid=d924b88500039398000000065aa492d5) | **Basic profiles** |
| **County Health Bureau** | Dr. A1 | Male, former Vice Director, responsible for public health | Dr. A2 | Male, Vice Director, responsible for public health |
|  | Dr. B1 | Male, Head, Department of Disease Control | Dr. B2 | Female, Head, Department of Disease Control |
| **County CDC** | Dr. C1 | Male, Vice director, responsible for TB control | Dr. C2^*^ | Male, Director of the CDC |
|  |  |  | Dr. D2 | Male, Vice director of the CDC, responsible for TB control |
|  | Dr. D1 | Male, Head, Department of TB Control | Dr. E2 | Male, Head, Department of TB Control |
|  | Dr. E1 | Male, TB control officer | Dr. F2 | Male, TB control officer |

^**^Interviewees invited through snow-balling; otherwise selected through purposive sampling.
