# Supplementary material for: ‘You say you are a TB doctor, but actually, you do not have any power’: health worker (de)motivation in the context of integrated, hospital-based tuberculosis care in eastern China
Source: Hum Resour Health. 2022 Jun 23;20:55. doi: 10.1186/s12960-022-00745-w (PMC9229519; doi:10.1186/s12960-022-00745-w)
Supplement: Supplementary file 2 — Additional file 2. List of interviewees in County A and County B (County Designated hospital). [file 12960_2022_745_MOESM2_ESM.docx]

Additional file 2 List of interviewees in County A and County B（County Designated hospital）

| **Departments** | **County A** | | **County B** | |
| --- | --- | --- | --- | --- |
|  | [**Pseudonym**](http://www.baidu.com/link?url=iGB0zWxS1Oos-FNfoBOWxCiQuZRwRklCrc7VyfMl10S52ADP2lYJaUZ5KL4UhBjWVRi3dADOaEDWujNcqJIiyqkvbfDF1M8jcdMZauj3KoK&wd=&eqid=d924b88500039398000000065aa492d5) | **Basic profiles** | [**Pseudonym**](http://www.baidu.com/link?url=iGB0zWxS1Oos-FNfoBOWxCiQuZRwRklCrc7VyfMl10S52ADP2lYJaUZ5KL4UhBjWVRi3dADOaEDWujNcqJIiyqkvbfDF1M8jcdMZauj3KoK&wd=&eqid=d924b88500039398000000065aa492d5) | **Basic profiles** |
| Management | Dr. F1 | Male, Vice Director | Dr. G2 | Male, Vice Director |
|  | Dr. G1^*^ | Male, Vice Director |  |  |
|  | Dr. H1 | Female, Head, Department of Public Health | Dr. H2 | Female, New Head, Department of Public Health |
|  |  |  | Dr. I2^*^ | Female, Former Head, Department of Public Health |
|  |  |  | Dr. J2 | Female, Staff. Department of Public Health |
|  | Dr. I1 | Male, Head, Department of Medical Affairs | Dr. K2 | Male, Head, Department of Medical Affairs |
|  | Dr. J1^*^ | Male, Head, Department of Accounting and Finance | Dr. L2^*^ | Female, Head, Department of Accounting and Finance |
| TB and infectious disease control department | Dr. K1 | Male, TB doctor, Head of TB Clinic | Dr.M2 | Female, TB doctor |
|  | Dr. L1 | Male, TB doctor, TB Clinic | Dr. N2 | Female, TB doctor |
|  | Dr. M1 | Female, Nursing staff (information), TB Clinic | Dr. O2 | Female, Nursing staff (information), TB Clinic |
|  | Dr. N1 | Female, Nursing staff (clinical), TB Clinic | Dr. P2 | Female, Nursing staff (clinical), TB Clinic |
|  | Dr. O1 | Male, Head, Department of Infectious Disease Control | Dr. Q2 | Female, Head, Department of Infectious disease Control |
|  | Dr. P1 | Female, Doctor, Department of Infectious Disease Control | Dr. R2^*^ | Female, Head, Department of Hospital Infection Management |
| Laboratory  TB registration  Registration | Dr. Q1 | Male, Staff with permanent contract, TB Laboratory | Dr. S2 | Female, Head, Central Laboratory |
|  | Dr. R1^*^ | Female, Staff without temporary contract, TB Laboratory | Dr. T2^*^ | Female, Staff, Central Laboratory, Rotated to TB Clinic |
|  | Ms. S1^*^ | Female, Staff, TB Clinic Registration Office | Dr. U2^*^ | Female, Staff, TB Clinic Registration Office |
| Pharmacists | Dr. T1^*^ | Female, Head, Central Pharmacy | Dr. V2^*^ | Male, Head, Central Pharmacy |
|  |  |  | Dr. W2 | Male, Head, TB pharmacy, Rotated |
|  | Dr. U1 | Male, Staff, TB pharmacy |  |  |
| Radiology | Dr. V1 | Male, Central Radiological Department | Dr. X2 | Male, Head of Central Radiological Department |
|  | Dr. W1^*^ | Male, Central Radiological Department |  |  |
| Number of interviewees |  | 18 |  | 18 |
|  |  |  |  |  |

^*^Interviewees invited through snow-balling; otherwise selected through purposive sampling.
