# Supplementary material for: ‘You say you are a TB doctor, but actually, you do not have any power’: health worker (de)motivation in the context of integrated, hospital-based tuberculosis care in eastern China
Source: Hum Resour Health. 2022 Jun 23;20:55. doi: 10.1186/s12960-022-00745-w (PMC9229519; doi:10.1186/s12960-022-00745-w)
Supplement: Supplementary file 3 — Additional file 3. Examples of interview topic guides. [file 12960_2022_745_MOESM3_ESM.docx]

**Additional file 3 Examples of interview topic guides**

**Managerial type of interview**

(Adapted for different managerial positions for health bureau, CDC and TB designated hospitals)

Profile

Can you please tell me a little about your background? (*probe: name, qualification, and years of experience; specific expertise in TB)*

- How long have you worked in the CDC (and worked with TB)?
- What are your main responsibilities? (list areas)
- Can you tell me roughly how much time you spend per week on these areas? (for each area listed)

Roles and responsibilities/division of labor

- What does the model of designated hospital mean for your responsibilities? And for the division of labor? (probe: *for the distribution of tasks and duties among staff responsible for TB services*)
- Can you describe the changes in your responsibilities since TB integration? Can you describe any changes in your workload since TB integration?

We notice the county government is paying more attention to public health work in the hospital and has given more and more public health work such as TB and other infectious control work to the hospital.

Organizational culture

- What does integration mean for communication across the staff responsible for TB services? How do you communicate with the hospital managers, health bureau and county government? How often?
- How do you feel about ownership of the program?
- What is the place of TB/designated hospital work among all the CDC work?
- Do you have regular meetings?
- How do the health bureau/county government respond if TB control/designated hospital work has difficulty? Can you give me an example? Are there examples of activities to promote team work?
- How do you supervise designated hospital’s work in TB? Can you give me an example? Do you think if it is effective not?
- What scale of attention do you receive from higher authorities (county government and health bureau, Ranking:1-5)? What are the top 3 priorities in the county level’s health agenda? Please explain – how and where is this reflected.

Resources

- What does this TB integration mean for resources, eg, human resource, infrastructure? Can you comment on the salary/ income structure of the designated hospital staff? as well as the human resource structure? Any preferential policy for the TB clinic and public health staff in the designated hospital?
- Do you feel the designated hospital has adequate staff to manage TB here?
- How about TB drugs and equipment? How are they purchased? Are there any problems? Do you think TB staff have adequate resources to use? Can you give me example of resource constraints? How does this influence clinical practice?

We have had the opportunity to reviewed about 200 patient charts. Could you comment on why doctors prescribe those drugs and examinations?

- Non-free drugs, i.e., first-line anti-TB drugs, liver protection drug, regulating immune medicine, gastrointestinal medicine and antibiotics
- CT, liver function and hepatitis test and others
- hospitalization

OVERALL – IN SUMMARY:

- What major challenges in managing TB in the designated hospital? What do you think designated hospital work vs. TB dispensary?
- What do you think are the main challenges for staff and patients?
- What are the main advantages of this approach for staff?....for patients?
- Do you have any suggestions for the future designated hospital TB work?
- What would improve your satisfaction working as X here to you?

**Practice type of interview**

(Adapted for TB clinical doctors, nurses and other health workers)

Profile

Can you please tell me a little about your background? (*probe: name, gender, qualification, and years of experience; specific expertise in TB)*

- How long have you worked in this clinic?
- What are your main responsibilities in this clinic? (list areas)
- Can you tell me roughly how much time you spend per week on these areas? (for each area listed)
- Did you receive specific training on TB services? If so, when? For how long? Can you recall up to 3 main issues that were the focus of the training?

Roles and responsibilities/division of labor under designated hospital

- When did you hear about the designated hospital/integration? how did you respond (what was your reaction?)?
- What does the model of designated hospital mean for your responsibilities? And for the division of labor?
- Can you describe the changes in your responsibilities since TB integration? Can you describe the workload since TB integration?

The government is paying more attention to public health work in the hospital and has given more and more public health work such as TB and other infectious control work to the hospital.

*Organizational culture*

- What does it mean for communication across the staff responsible for TB services? How do you communicate with the hospital mangers? How do you communicate with public health department, infectious disease department, and other staffs ( CDC staff )? How do the hospital leaders respond if your department has difficulty? Can you give me an example? Are there examples of activities to promote team work?
- Who supervises your work? How are you supervised? Do you think if it is effective not?
- What motivates you in your work? What demotivates you?
- What scale of attention do you receive from higher authorities(hospital directors, health bureau, CDC)? Please explain – how and where is this reflected.
- What are the top 3 priorities in the hospital? How is reflected in TB clinic? How do you feel about ownership of the program? Do you have regular meetings?

Resources

- Can you tell me about the salary/ income structure? How does the hospital allocate the salary and bonus for TB clinic staff and yourself? What is operational mode of TB clinic staff (rotation, temporary…)? Any preferential policy for TB clinic and public health staff?
- Do you feel there are adequate staff to manage TB here?
- How about TB drugs and equipment? How are they purchased? Are there any problems? Do you have adequate resources? Can you give me example of resource constraints? How does this influence clinical practice?

Clinical management and case review

- What are the main differences in clinical management procedures as compared with managing TB at the TB dispensary? Probe: resources, procedures, guidelines, quality control…..
- Who do you work closely with in managing patient? When is there a need to communicate with other staff involved in management of the patient e.g. nurse, pharmacists, lab technician?
- How do you communicate with them? Can you give me a recent example of a patient where you had to confer with others regarding management?

With your permission, can we discuss some of your cases? Look at register….and select 4 to 5 cases, ideally managed within last 2 weeks – ask about:

- Tell me a bit about this patient….profile etc
- Patient pathway including any delays, diagnostic process, treatment and prescriptions
- What influenced decisions? E.g., profiles of patients? stages of illnesses?
- What was prescribed and why?
- Anything unusual about this patient?

Thank you. We had the opportunities to reviewed 200 patient charts – the data showed x and y. Could you comment on why doctors prescribe the drugs and examinations as below?

- Non-free drugs, i.e., first-line anti-TB drugs, liver protection drug, regulating immune medicine, gastrointestinal medicine and antibiotics
- CT, liver function and hepatitis test and others
- hospitalization

OVERALL – IN SUMMARY:

- What major challenges in managing TB in this hospital? What do you think designated hospital work vs. TB dispensary?
- What do you think are the main challenges for staff and patients?
- What are the main advantages of this approach for staff?....for patients?
- Do you have any suggestions for the future designated hospital TB work?
- What would improve your job satisfaction to you?
