# Supplementary material for: ‘You say you are a TB doctor, but actually, you do not have any power’: health worker (de)motivation in the context of integrated, hospital-based tuberculosis care in eastern China
Source: Hum Resour Health. 2022 Jun 23;20:55. doi: 10.1186/s12960-022-00745-w (PMC9229519; doi:10.1186/s12960-022-00745-w)
Supplement: Supplementary file 4 — Additional file 4. COREQ (COnsolidated criteria for REporting Qualitative research) checklist. [file 12960_2022_745_MOESM4_ESM.doc]

**COREQ** **(COnsolidated** **criteria** **for** **REporting** **Qualitative** **research)** **Checklist**

| **Topic** | **Item** **No.** | **Guide** **Questions/Description** | **Reported** **on** **Page** **No.** |
| --- | --- | --- | --- |
| **Domain** **1:** **Research** **team**  **and** **reﬂexivity** | | | |
| Personal characteristics | | | |
| Interviewer/facilitator | 1 | Which author/s conducted the interview or focus group? | 7 |
| Credentials | 2 | What were the researcher’s credentials? E.g. PhD, MD | 7-8 |
| 56Occupation | 3 | What was their occupation at the time of the study? | 7-8 |
| Gender | 4 | Was the researcher male or female? | 7 |
| Experience and training | 5 | What experience or training did the researcher have? | 7-8 |
| *Relationship* *with*  *participants* | | | |
| Relationship established | 6 | Was a relationship established prior to study commencement? | 8 |
| Participant knowledge of the interviewer | 7 | What did the participants know about the researcher? e.g. personal goals, reasons for doing the research | 8 |
| Interviewer characteristics | 8 | What characteristics were reported about the inter viewer/facilitator? e.g. Bias, assumptions, reasons and interests in the research topic | 8，21 |
| **Domain** **2:** **Study** **design** | | | |
| *Theoreticalframework* | | | |
| Methodological orientation and Theory | 9 | What methodological orientation was stated to underpin the study? e.g. grounded theory, discourse analysis, ethnography, phenomenology, content analysis | 9 |
| *Participant* *selection* | | | |
| Sampling | 10 | How were participants selected? e.g. purposive, convenience, consecutive, snowball | 8 |
| Method of approach | 11 | How were participants approached? e.g. face-to-face, telephone, mail, email | 8 |
| Sample size | 12 | How many participants were in the study? | 8 |
| Non-participation | 13 | How many people refused to participate or dropped out? Reasons? | n/a |
| *Setting* | | | |
| Setting of data collection | 14 | Where was the data collected? e.g. home, clinic, workplace | 8-9 |
| Presence of non-  participants | 15 | Was anyone else present besides the participants and researchers? | 9 |
| Description of sample | 16 | What are the important characteristics of the sample? e.g. demographic data, date | 8 |
| *Data* *collection* | | | |
| Interview guide | 17 | Were questions, prompts, guides provided by the authors? Was it pilot tested? | 8 |
| Repeat interviews | 18 | Were repeat inter views carried out? If yes, how many? | n/a |
| Audio/visual recording | 19 | Did the research use audio or visual recording to collect the data? | 9 |
| Field notes | 20 | Were ﬁeld notes made during and/or after the inter view or focus group? | 9 |
| Duration | 21 | What was the duration of the inter views or focus group? | 9 |
| Data saturation | 22 | Was data saturation discussed? | 8 |
| Transcripts returned | 23 | Were transcripts returned to participants for comment and/or correction? | 10 |

| **Topic** | **Item** **No.** | **Guide** **Questions/Description** | **Reported** **on** **Page** **No.** |
| --- | --- | --- | --- |
| **Domain** **3:** **analysis** **and**  **ﬁndings** | | | |
| *Data* *analysis* | | | |
| Number of data coders | 24 | How many data coders coded the data? | 10 |
| Description of the coding tree | 25 | Did authors provide a description of the coding tree? | 9 |
| Derivation of themes | 26 | Were themes identiﬁed in advance or derived from the data? | 9 |
| Software | 27 | What software, if applicable, was used to manage the data? | 9 |
| Participant checking | 28 | Did participants provide feedback on the ﬁndings? | 10 |
| *Reporting* | | | |
| Quotations presented | 29 | Were participant quotations presented to illustrate the themes/ﬁndings? Was each quotation identiﬁed? e.g. participant number | 10-17 |
| Data and ﬁndings consistent | 30 | Was there consistency between the data presented and the ﬁndings? | 10-17 |
| Clarity of major themes | 31 | Were major themes clearly presented in the ﬁndings? | 10-17 |
| Clarity of minor themes | 32 | Is there a description of diverse cases or discussion of minor themes? | 10-17 |

Developed from: Tong A, Sainsbury P, Craig J. Consolidated criteria for reporting qualitative research (COREQ): a 32-item checklist

for interviews and focus groups. *International* *Journalfor* *Quality* *in* *Health* *Care* . 2007. Volume 19, Number 6: pp. 349 – 357
